# The Role of Healthcare-Related Experiences in Willingness and Preference for Long-Acting Injectable PrEP (LAI-PrEP) Among Transfeminine People in the United States

**DOI:** 10.1007/s10461-025-05016-y

**Published:** 2026-01-27

**Authors:** Jennifer L. Glick, Danielle F. Nestadt, Travis Sanchez, Irah L. Lucas, Joanna A. Caldwell, Mariah Valentine-Graves, Savannah Winter, Duygu Islek, Sarah M. Murray, Stefan Baral, Kimberley Brown, Leigh Ragone, Annemiek de Ruiter, Supriya Sarkar, Vani Vannappagari

**Affiliations:** 1 Community Health Sciences and Policy School of Public Health, Louisiana State University Health Science Center, New Orleans, LA 70112-2272 USA; 2 Department of Health, Behavior and Society, Johns Hopkins Bloomberg School of Public Health, Baltimore, MD USA; 3 Department of Epidemiology, Emory Rollins School of Public Health, Atlanta, GA USA; 4 Department of Mental Health, Johns Hopkins Bloomberg School of Public Health, Baltimore, MD USA; 5 Department of Epidemiology, Johns Hopkins Bloomberg School of Public Health, Baltimore, MD USA; 6 ViiV Healthcare, Durham, NC USA

**Keywords:** Transgender women and transfeminine people (TWTFP), PrEP, HIV risk, Stigma, Healthcare providers

## Abstract

Transgender women and transfeminine people (TWTFP) experience disproportionately high HIV prevalence, low HIV pre-exposure prophylaxis (PrEP) coverage, and negative healthcare experiences. Long-acting injectable PrEP (LAI-PrEP) and daily oral-PrEP are delivered under different healthcare interaction protocols. We examined the role of healthcare-related experiences on willingness and preference for LAI-PrEP among a U.S. nationwide sample of sexually active TWTFP aged 15+. Recruitment occurred between 6/2022 and 10/2023 via social media advertisements for a cross-sectional online sexual health survey. Analyses included past-year PrEP naïve TWTFP with no prior HIV diagnosis (*N* = 1648). Participants reported LAI-PrEP willingness and ranked PrEP modality preferences; recent healthcare-related experiences were explored as correlates, using adjusted multivariable Poisson regression with robust variance estimation. Among respondents, 26.3% were willing to use LAI-PrEP (*n* = 433/1648). Among participants willing to use LAI-PrEP and another modality (*n* = 390/433; 90.1%), 45.6% (*n* = 178/390) preferred LAI-PrEP. Discussing sexual health with a healthcare provider (HCP) was associated with increased LAI-PrEP willingness (adjusted prevalence ratio [aPR] = 1.34; 95% confidence interval [CI] = 1.12–1.60; *p* = 0.001); use of oral prescription medication (non-hormonal) was associated with decreased LAI-PrEP willingness (aPR = 0.60; 95% CI = 0.38–0.93; *p* = 0.022). No significant associations were found between healthcare-related experiences and LAI-PrEP preference. Given that most TWTFP who were willing to use LAI-PrEP were also willing to use oral-PrEP, but nearly half preferred LAI-PrEP, offering multiple PrEP modalities is essential to meet their needs. Associations between sexual health discussions and increased LAI-PrEP willingness highlight the importance of HCP engagement; initiatives which encourage HCPs to initiate LAI-PrEP conversations in supportive ways are crucial. Associations between oral prescription use and decreased LAI-PrEP willingness, coupled with null findings related to injection use, highlight the need for further investigation.

**Supplementary Information:**

The online version contains supplementary material available at https://doi.org/10.1007/s10461-025-05016-y.

## Resumen

Las mujeres transgénero y las personas transfemeninas (TWTFP) experimentan una prevalencia desproporcionadamente alta de VIH, baja cobertura de profilaxis previa a la exposición (PrEP) y experiencias negativas en el sistema de salud. La PrEP inyectable de acción prolongada (LAI-PrEP) y la PrEP oral diaria se administran bajo diferentes protocolos de interacción en la atención médica. Examinamos las experiencias relacionadas con la atención en la disposición y preferencia por LAI-PrEP en una muestra nacional de TWTFP sexualmente activas mayores de 15 años en EE. UU. El reclutamiento tuvo lugar del 06/2022 a 10/2023 a través de anuncios en redes sociales para una encuesta transversal sobre salud sexual. Se incluyeron TWTFP no expuestas a PrEP en el último año, y sin diagnóstico previo de VIH (*N* = 1648). Los participantes informaron su disposición a usar LAI-PrEP y clasificaron sus preferencias de modalidad de PrEP; se exploraron experiencias recientes en atención médica como correlatos, utilizando regresión de Poisson multivariable ajustada. Entre los encuestados, el 26.3% estaba dispuesto a usar LAI-PrEP (*n* = 433/1648). Entre quienes estaban dispuestos a usar LAI-PrEP y otra modalidad (*n* = 390/433; 90.1%), el 45.6% (*n* = 178/390) prefería LAI-PrEP. Hablar de salud sexual con un proveedor de atención médica (PS) se asoció con una mayor disposición a usar LAI-PrEP (razón de prevalencia ajustada [aPR] = 1.34; IC del 95%=1.12–1.60; *p* = 0.001); el uso de medicamentos recetados orales (no hormonales) se asoció con menor disposición a usar LAI-PrEP (aPR = 0.60; IC del 95%=0.38–0.93; *p* = 0.022). No se encontraron asociaciones significativas entre experiencias en atención y preferencia por LAI-PrEP. Dado que la mayoría de las TWTFP dispuestas a usar LAI-PrEP también estaban dispuestas a usar PrEP oral, ofrecer múltiples modalidades de PrEP es esencial. Las asociaciones entre las conversaciones sobre salud sexual y la mayor disposición a usar LAI-PrEP destacan la importancia de la participación de los PS; son fundamentales las iniciativas que alienten a los PS a iniciar conversaciones sobre LAI-PrEP. Las relaciones entre el uso de medicamentos orales y la disminución de la disposición hacia la LAI-PrEP, junto con la ausencia de hallazgos significativos sobre el uso de inyecciones, subrayan la necesidad de realizar más investigaciones.

## Introduction

Transgender women and transfeminine people (TWTFP) experience disproportionately high HIV rates; TWTFP accounted for 2% of new HIV infections in the United States (U.S.) in 2021 despite representing only 0.23% of the general population [1, 2]. Oral HIV pre-exposure prophylaxis (PrEP) was first approved by the U.S. Food and Drug Administration (FDA) in 2012 as a safe and effective method of preventing HIV infection [3]. Rates of PrEP awareness among TWTFP in the U.S. are relatively high, with notable increases in recent years [4, 5]. One study showed that approximately half of TWTFP had spoken to their provider about PrEP [5]. However, initiation rates remain low; estimates among TWTFP range from 3% to 32% [5–8].

Known barriers to oral-PrEP uptake among TWTFP include gender-based and HIV/PrEP- related stigma from healthcare providers and/or close social networks, distrust of healthcare providers and systems, health insurance coverage barriers, and difficulty navigating financial assistance programs [9–14]. TWTFP experience pervasive stigma and negative healthcare experiences, which limit their access to various HIV prevention and healthcare resources [15–17]. Stigma, a process of labeling, stereotyping, separation, status loss, and discrimination in a context in which power is exercised [18], manifests for affected individuals in multiple ways. Enacted stigma refers to experiences of unfair treatment and violence, whereas anticipated stigma is the expectation of rejection or mistreatment. In one study, a quarter to nearly half of TWTFP reported past experiences of discrimination and reported delaying preventive care due to fear of discrimination [15]. Discrimination in health and non-health settings and different types of discrimination—being denied services, verbally harassed, or physically assaulted—were all significantly associated with delaying care; discrimination at a health location had the strongest association with care postponement [15]. Further, stigmatizing interactions with healthcare providers driven by transphobia, both in general and while accessing PrEP services, discourage continued engagement with the healthcare system [13]. In addition, word-of-mouth sharing of negative experiences may foster medical mistrust among TWTFP communities, creating a reluctance to initiate PrEP (e.g., anticipated stigma) for fear of mistreatment [14]. Conversely, access to gender-affirming healthcare providers is positively associated with PrEP use among TWTFP [19].

Injectable cabotegravir, a long-acting form of injectable PrEP (LAI-PrEP), received FDA approval in December 2021 for use with adolescents and adults at risk of HIV [20]. Cabotegravir for LAI-PrEP has been shown to be safe and effective in preventing HIV among TWTFP [21]. LAI-PrEP holds the potential to overcome various uptake and adherence-related challenges of daily oral-PrEP [22–25], such as providing a greater level of discretion (as no pills are required) and minimizing exposure to PrEP-related stigma [26, 27].

LAI-PrEP offers an alternative healthcare interaction protocol from oral-PrEP [28]. Standard healthcare interaction protocols for oral-PrEP include quarterly follow-up visits with a healthcare provider for behavioral risk reduction support and to monitor HIV status, bacterial STIs, and medication adherence, with additional tests (e.g., renal function) at 6- and 12-month appointments [28]. Current federal LAI-PrEP protocol guidance indicates administration by a healthcare provider, including an initial shot, a one-month follow up shot, and then administration every 2 months through gluteal intramuscular injection; HIV and STI testing are to be conducted at various intervals during the subsequent 2-month injection visits [28]. Implementation protocols are still being developed concerning ideal location and provider-type for LAI-PrEP injections and related tests, such that a patient might see different provider types at different types of facility (e.g., pharmacy) for prescriptions, testing, and injections [29, 30]. While LAI-PrEP may require more healthcare interactions than oral-PrEP, they may be at different healthcare locations with different types of healthcare providers.

LAI-PrEP holds the potential to improve PrEP uptake and retention among TWTFP. An emerging body of literature has demonstrated high levels of interest in and preference for LAI-PrEP among both PrEP-experienced and PrEP-naïve TWTFP, including TWTFP at highest risk for HIV infection [31, 32]. While data concerning TWTFP’s preferences for initiating PrEP across different PrEP-modality options is becoming available [33, 34], the role of past healthcare-related experiences (e.g., stigma-related, accessing care, etc.) has yet to be explored. This is an important gap, given known associations between healthcare-related experiences and other health-seeking behaviors and health outcomes among this population. Therefore, we examined the role of past healthcare-related experiences on willingness and preference for LAI-PrEP among a U.S. nationwide online sample of TWTFP.

## Methods

### Study Design and Analytical Sample

We used data from the 2022 U.S. Transgender Women’s Internet Survey and Testing (TWIST) study, which surveyed TWTFP using online recruitment and data collection between June 2022 and October 2023. The recruitment and data collection methods for TWIST have been reported previously [35]. Briefly, key study implementation decisions were informed and reviewed by a community advisory board. Recruitment included advertisements on a variety of websites, social media, and dating apps. Participants were eligible if they resided in the U.S., were 15 years or older, currently identified as women or transgender women/transfeminine, reported male sex at birth, and reported at least one experience of oral, anal, or vaginal sex in their lifetime. Eligible TWTFP who provided informed consent completed the survey online in English or Spanish, which included questions about demographic characteristics; sexual and drug-use behaviors; HIV & STI prevention activities, including PrEP; healthcare utilization; and social determinants of health. Half of the sample was randomized to receive a set of gender identity-related stigma questions adapted from a sexual behavior stigma measure previously validated with men who have sex with men [36]. Participants who completed the survey received a $10 e-gift card. Activities were approved by the [REDACTED] institutional review board.

Analyses were restricted to participants who did not report prior HIV diagnosis, had not taken any PrEP modality in the past year, responded to a survey item about willingness to use LAI-PrEP, and were randomized to receive questions about gender identity-related stigma.

### Measures

#### Outcome Variables

**Fig. 1:**
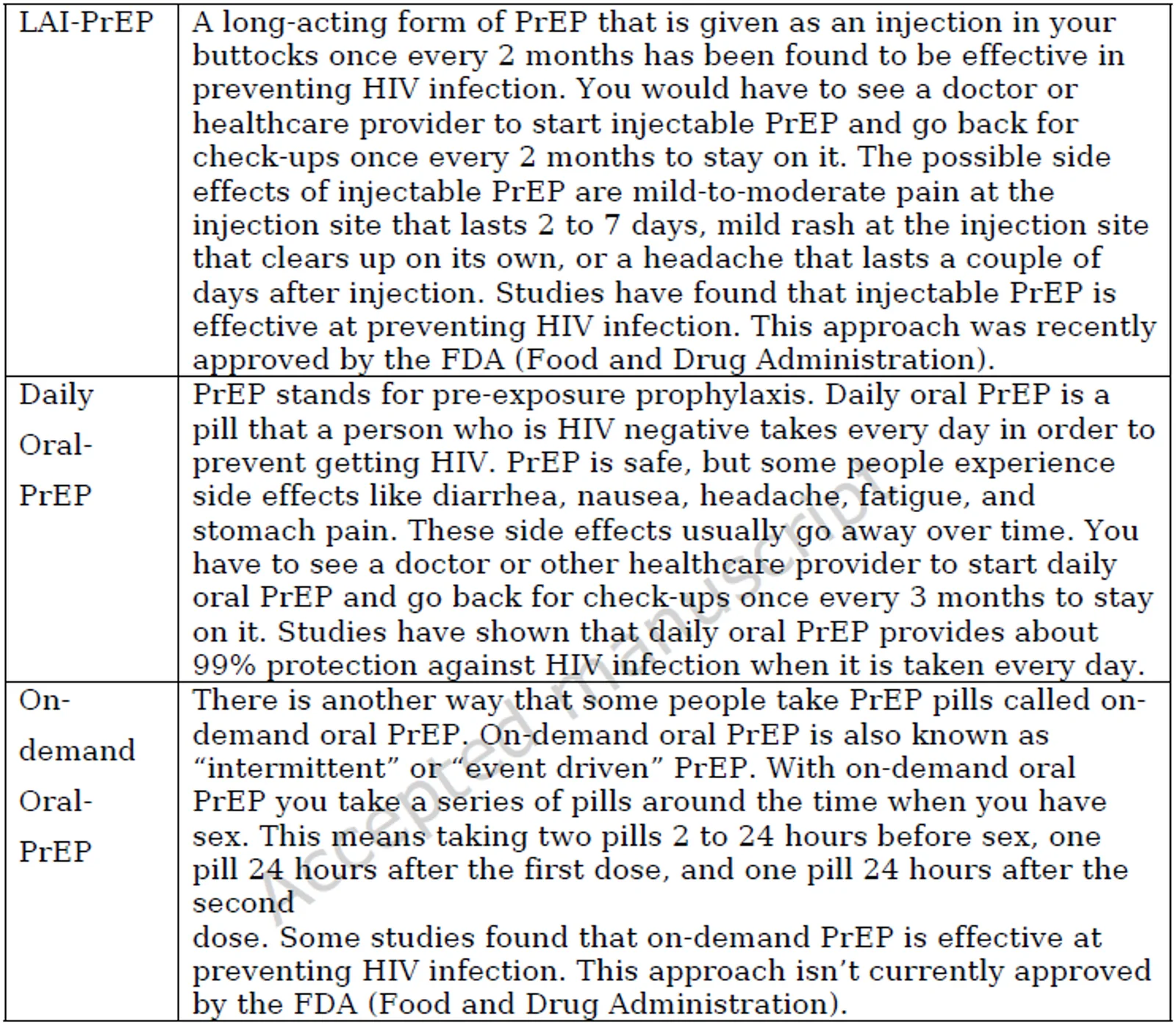
Background information presented about each PrEP modality in TWIST survey

Participants were presented with a brief description of three PrEP modalities —daily oral, on-demand, and LAI-PrEP (see Fig. 1)—before being asked, “If [this modality of] PrEP were available from your local doctor and you could access it for free, would you go to your doctor in the next month to start [this modality of] PrEP?” Those who responded “Yes” to the LAI-PrEP item were considered **willing to use LAI-PrEP.** Participants who were willing to use LAI-PrEP and at least one of the oral modalities were asked to rank their preferences; those who ranked LAI-PrEP first were categorized as **preferring LAI-PrEP**.

#### Explanatory Variables

50% of the total TWIST sample was randomized to receive **gender-related healthcare stigma**items on their surveys. Healthcare-related gender identity stigma, known to impact HIV prevention service use among this population [37], was measured using a modified existing measure [36, 37]. Our measure included items assessing feelings, behaviors, and interactions the participant experienced *because of their gender identity*. Participants reported whether they had experienced each item within the past year. A mean score on five items related to **anticipated gender-related healthcare stigma** (e.g., “Have you ever avoided going to health care services because you worry someone may treat you poorly because of your gender identity?”; Supplementary Table 1: Gender-related Healthcare Stigma Items) was calculated, and the score was dichotomized at the sample median to yield high vs. low anticipated gender-related healthcare stigma. Sensitivity analyses were conducted using various cut-points to dichotomize the stigma score and informed the selection to dichotomize at the median (Supplementary Table 2).

If participants endorsed either of two items related to past-year enacted gender-related healthcare stigma (i.e., “Have you heard healthcare providers gossiping about you (talking about you) because of your gender identity?” and “Have you felt that you were not treated well in a health center because someone knew about your gender identity?”), they were categorized as having experienced **enacted gender-related healthcare stigma** in the past year.

We measured two aspects of **gender-affirming healthcare use**, past year use of hormones or hormone blockers for medical transition or gender affirmation (yes vs. no) and any history of gender-affirming surgery (none vs. any genital surgery vs. only non-genital surgery).

As a dimension of **healthcare communication**, we assessed whether the participant had seen a healthcare provider who talked to them about sex/sexual health in the past year.

**Medication modality use/experience (non-hormonal)** was assessed with items asking whether participants were currently taking daily prescription pills and whether they had any non-vaccine injection of prescription medication in the past year. We constructed a variable to capture non-hormonal use of medication by pill and by injection (no use, pill only use, injection only use, and both pill and injection use). Participants who reported hormone use by a modality they also reported using for prescription medications were categorized as “no use” in this variable to limit multicollinearity with the hormone use variable described above.

#### Additional covariates

Adjusted models controlled for socio-demographic and behavioral variables that were selected a priori based on previous significant findings of association with willingness to use LAI-PrEP among TWTFP in the TWIST sample or with PrEP willingness and preference more broadly [34, 38, 39]. These include age in years (15–24, 25–29, 30–39, 40+), race/ethnicity (non-Hispanic Black, non-Hispanic white, Hispanic of any race, or “Other/multiple/unknown”), U.S. Census region of residence (Northeast, Midwest, South, or West – based on self-reported ZIP code), health insurance type (private only, public only, both private and public, other, no insurance), past-year condomless anal sex (yes/no), number of sex partners (one/two or more), any illicit non-injection drug use other than marijuana (yes/no), and prior awareness of LAI-PrEP (“*Before today*,* have you ever heard of LAI-PrEP?*”; yes/no).

### Statistical Analysis

Two different outcomes were explored in this study, each in its own model. For analyses examining overall willingness to use LAI-PrEP, the analytic sample included participants who did not report prior HIV diagnosis, had not taken any PrEP modality in the past year, responded “yes,” “no,” or “don’t know” to the item about willingness to use LAI-PrEP, and received questions about gender-related stigma. For analyses examining a preference for LAI-PrEP over other modalities, the sample was further restricted to participants who were willing to use LAI-PrEP and at least one of the oral modalities.

We measured the prevalence of each explanatory variable within each sample, stratified by each outcome, and used Pearson’s χ2 tests to test for significant differences. We used bivariate and multivariable Poisson regression with robust variances to estimate prevalence ratios (PR) and 95% confidence intervals (CI). Robust Poisson method results can be interpreted as risk or prevalence ratios when modeling a binary outcome, even when the outcome is not rare, unlike logistic regression modeling [40]. Regression models were adjusted for age, race/ethnicity, census region, type of health insurance, condomless anal sex in past year, multiple sexual partners in past year, illicit drug use in past year, and prior awareness of LAI-PrEP. All analyses were conducted using Stata/MP 15.1 (Stata Corp, College Station, TX).

## Results

### Sample Description

Participants (*N* = 1648) were HIV-negative, had not used PrEP in the past year, and were randomized to receive the stigma items used in analyses (Table 1). The majority (73.4%) were non-Hispanic White, more than one-third (36.8%) were 15–24 years old, and about one-third (31.9%) lived in the South. About half (50.8%) reported high levels of anticipated gender-related healthcare stigma and a quarter (25.1%) had experienced enacted gender-related healthcare stigma in the past year. Nearly three-quarters (72.3%) reported past-year use of hormones/hormone blockers for gender affirmation, while nearly one-third (31.6%) reported a history of gender-affirming surgery, among whom 46.3% reported genital surgery. Approximately half (49.5%) had discussed sex/sexual health with an HCP in the past year. Past-year non-hormonal medication modality use by pill only, injection only, and both pill and injection were reported by 8.9%, 1.5%, and 2.6% of participants, respectively.

**Table 1:** Sample characteristics of participants (*N* = 1648)^a^ in the transgender women’s internet survey and testing project, 2022–2023

	*n*	%
***Healthcare-related experiences*** ^b^		
High anticipated gender-related healthcare stigma (vs. low)	833	50.8%
Any enacted gender-related healthcare stigma	407	25.1%
Use of hormones/hormone blockers	1186	72.3%
*History of gender-affirming surgery* ^c^		
None	1125	68.4%
Any genital surgery	241	14.6%
Only non-genital surgery	279	17.0%
Discussed sex/sexual health with HCP	802	49.5%
*Medication modality use (non-hormonal)*		
No use	1428	87.0%
Pill use only	146	8.9%
Injection use only	25	1.5%
Pill & injection use	42	2.6%
***Additional covariates***		
*Age*		
15–24	606	36.8%
5–29	352	21.4%
30–39	407	24.7%
40+	283	17.2%
*Race/ethnicity*		
Black, non-Hispanic	103	6.3%
Hispanic	159	9.7%
White, non-Hispanic	1209	73.4%
Other/multiple/unknown	177	10.7%
*U.S. census region of residence*		
Northeast	284	17.2%
Midwest	356	21.6%
South	526	31.9%
West	482	29.3%
*Health insurance*		
None	126	7.8%
Private only	1021	63.1%
Public only	344	21.3%
Other/multiple	127	7.9%
Condomless anal sex, past year	557	34.1%
2 + sexual partners, past year	647	40.0%
Illicit drug use, past year	384	23.3%
Prior awareness of LAI-PrEP	335	21.3%

About one-quarter of the sample was willing to use LAI-PrEP (*n* = 433/1648; 26.3%). Among LAI-PrEP willing participants, most (*n* = 390/433; 90.1%) were willing to use both LAI-PrEP and one of the oral modalities; one in ten TWTFP were only willing to use LAI-PrEP and no other modalities. Finally, among those willing to use both LAI-PrEP and an oral modality, nearly half preferred LAI-PrEP (*n* = 178/390; 45.6%).

### Willingness to Use LAI-PrEP

In unadjusted models (Table 2), past-year enacted gender-related healthcare stigma (prevalence ratio [PR] = 1.26; 95% confidence interval [CI] = 1.06–1.50; *p* = 0.010), discussion of sex/sexual health with a HCP (PR = 1.39; 95% CI = 1.18–1.64; *p* < 0.001), past-year pill medication modality use (PR = 0.64; 95% CI = 0.44–0.92; *p* = 0.017), and past-year injection medication modality use (PR = 1.79; 95% CI = 1.18–2.72; *p* = 0.006) were significantly associated with willingness to use LAI-PrEP.

**Table 2:** Healthcare-related experiences and willingness to use LAI-PrEP among TWIST participants (*n* = 1648)^a^

	Not willing to use LAI-PrEP or not sure *n* (%)	Willing to use LAI-PrEP *n* (%)	Unadjusted Prevalence Ratio (95% confidence interval)	*p*-value	Adjusted^b^ Prevalence Ratio (95% confidence interval)	*p*-value
Total	1215 (73.7%)	433 (26.3%)				
Anticipated gender-related healthcare stigma, high vs. low^c^	603 (72.4%)	230 (27.6%)	1.12 (0.95–1.31)	0.189	1.11 (0.93–1.33)	0.234
Any enacted gender-related healthcare stigma^c^	281 (69.0%)	126 (31.0%)	1.26 (1.06–1.50)	0.010*	1.05 (0.87–1.27)	0.616
Use of hormones/hormone blockers^c^	865 (72.9%)	321 (27.1%)	1.13 (0.93–1.36)	0.213	0.95 (0.75–1.19)	0.644
History of gender-affirming surgery^d^						
None	826 (73.4%)	299 (26.6%)	Ref.		Ref.	
Any genital surgery	181 (75.1%)	60 (24.9%)	0.94 (0.74–1.19)	0.593	0.94 (0.74–1.20)	0.645
Only non-genital surgery	205 (73.5%)	74 (26.5%)	1.00 (0.80–1.24)	0.985	0.96 (0.77–1.19)	0.693
Discussed sex/sexual health with HCP^c^	556 (69.3%)	246 (30.7%)	1.39 (1.18–1.64)	< 0.001*	1.34 (1.12–1.60)	0.001*
Prescription medication modality use (non-hormonal)^c^						
None	1045 (73.2%)	383 (26.8%)	Ref.		Ref.	
Pill only use	121 (82.9%)	25 (17.1%)	0.64 (0.44–0.92)	0.017*	0.60 (0.38–0.93)	0.022*
Injection only use	13 (52.0%)	12 (48.0%)	1.79 (1.18–2.72)	0.006*	1.16 (0.64–2.08)	0.624
Pill & injection use	29 (69.1%)	13 (30.9%)	1.15 (0.73–1.83)	0.541	0.90 (0.58–1.41)	0.655

^a^Data for some variables does not add up to the total number of participants due to missing information resulting from non-response from some of the participants

^b^Adjusted for all variables in table, as well as age, race/ethnicity, census region, type of health insurance, condomless anal sex in past year, multiple sexual partners in past year, illicit drug use in past year, and prior awareness of LAI-PrEP

^c^Past year

^d^ Ever

*p < 0.05

In adjusted models, discussion of sexual health with a HCP remained positively associated with willingness to use LAI-PrEP (adjusted PR [aPR] = 1.34; 95% CI = 1.12–1.60; *p* = 0.001), and past-year use of oral medicine remained negatively associated with willingness to use LAI-PrEP (aPR = 0.60; 95% CI = 0.38–0.93; *p*= 0.022). Mean variance inflation factor (VIF) for the adjusted model was 2.49, indicating an acceptable level of multicollinearity [41], with individual VIFs suggesting collinearity primarily related to control variables.

### Preference for LAI-PrEP

In unadjusted models (Table 3), among participants who were willing to use both LAI-PrEP and an oral-PrEP modality, past-year use of hormones/hormone blockers (PR = 1.53; 95% CI = 1.13–2.07; *p* = 0.006) and history of non-genital gender-affirming surgery (PR = 1.26, 95% CI = 1.07–1.72; *p*= 0.011) were associated with preference for LAI-PrEP. Neither of these associations remained statistically significant in adjusted models. Mean VIF for the adjusted model was 2.44, indicating an acceptable level of multicollinearity [41], with individual VIFs suggesting collinearity primarily related to control variables.

**Table 3:** Healthcare-related experiences and preference for LAI-PrEP over oral modalities among TWIST participants who were willing to use LAI-PrEP and at least one other modality (*n* = 390)^a^

	Prefer daily oral or on-demand *n* (%)	Prefer LAI-PrEP *n* (%)	Unadjusted Prevalence Ratio (95% confidence interval)	*p*-value	Adjusted^b^ Prevalence Ratio (95% confidence interval)	*p*-value
Total	212 (54.4%)	178 (45.6%)				
Anticipated gender-related healthcare stigma, high vs. low^c^	111 (53.6%)	96 (46.4%)	1.03 (0.83–1.28)	0.787	1.03 (0.82–1.30)	0.780
Any enacted gender-related healthcare stigma^c^	69 (59.0%)	48 (41.0%)	0.86 (0.67–1.11)	0.249	0.92 (0.71–1.19)	0.522
Use of hormones/hormone blockers^c^	142 (49.5%)	145 (50.5%)	1.53 (1.13–2.07)	0.006*	1.38 (0.95–1.99)	0.090
History of gender-affirming surgery^d^						
None	149 (55.4%)	120 (44.6%)	Ref.		Ref.	
Any genital surgery	37 (67.3%)	18 (32.7%)	0.73 (0.49–1.10)	0.131	0.69 (0.45–1.06)	0.088
Only non-genital surgery	26 (39.4%)	40 (60.6%)	1.26 (1.07–1.72)	0.011*	1.08 (0.83–1.40)	0.551
Discussed sex/sexual health with HCP^c^	123 (55.2%)	100 (44.8%)	0.99 (0.79–1.24)	0.923	0.89 (0.70–1.13)	0.337
Prescription medication modality (non-hormonal)^c^						
None	183 (52.7%)	164 (47.3%)	Ref.		Ref.	
Pill only use	13 (61.9%)	8 (38.1%)	0.81 (0.46–1.41)	0.448	1.43 (0.78–2.62)	0.242
Injection only use	6 (60.0%)	4 (40.0%)	0.85 (0.39–1.82)	0.670	0.54 (0.08–3.61)	0.529
Pill & injection use	10 (83.3%)	2 (16.7%)	0.35 (0.10–1.26)	0.108	0.46 (0.14–1.52)	0.202

^a^Data for some variables does not add up to the total number of participants due to missing information resulting from non-response from some of the participants

^b^Adjusted for all variables in table, as well as age, race/ethnicity, census region, type of health insurance, condomless anal sex in past year, multiple sexual partners in past year, illicit drug use in past year, and prior awareness of LAI-PrEP

^c^Past year

^d^Ever

*p < 0.05

## Discussion

We examined the role of past healthcare-related experiences on willingness and preference for LAI-PrEP among a U.S. nationwide online sample of TWTFP at risk of HIV who are not currently using PrEP. Just over a quarter of respondents were willing to use LAI-PrEP; having had a sexual health discussion with an HCP increased LAI-PrEP willingness, and past-year use of oral prescription medication decreased LAI-PrEP willingness. While many of the healthcare-related experiences we examined, including stigma, were not associated with LAI-PrEP willingness and preference in adjusted models, our findings reveal critical insights. Given the disproportionate burden of HIV among TWTFP, all insights related to increasing prevention activities are useful to inform enhanced HIV prevention efforts with this population.

We found that among those willing to use LAI-PrEP, one in ten TWTFP were only willing to use LAI-PrEP and no other modalities. Nearly half (45.6%) of the participants who were willing to use LAI-PrEP and an oral modality preferred LAI-PrEP. However, our examination into healthcare-related experiences associated with these preferences yielded null findings; no significant associations were found in adjusted models between healthcare-related experience variables of interest and preference for LAI-PrEP. It is worth noting that at the bivariate level, we found that past-year use of hormones/hormone blockers and history of non-genital gender-affirming surgery was positively associated with preference for LAI-PrEP. While this did not remain significant in adjusted models, it may still suggest a preference for LAI-PrEP among those engaged with gender-affirming healthcare, especially given literature which indicates that co-locating PrEP services with gender-affirmation care may support PrEP use [42, 43]. Therefore, engagement efforts between HCPs and TWTFP patients may promote PrEP uptake, especially LAI-PrEP. Future research could further examine this topic, including additional explanatory variables, interaction effects, and subgroup analyses. Past medication modality experiences were relevant, as use of oral prescription medication decreased willingness to use LAI-PrEP. Notably, use of injection prescription medication was not associated with LAI-PrEP willingness or preference in adjusted models, but it was significantly associated with willingness at the bivariate level. While these findings do not provide a conclusive understanding, they suggest that people may be less comfortable with a medication modality they do not already use.

These preliminary modality findings must be considered in light of several factors. First, given multicollinearity concerns between variables of interest and limitations in the wording of the survey items, our modality variable was limited to prescription medications excluding hormones and vaccines. Future modality experience research should utilize survey items that allow for a more comprehensive modality experience to be captured. Further, given that the outcome variable includes initiating LAI-PrEP in the coming 30 days, the hesitation could be around the timeframe of adding in a new medication modality. Common concerns about possible interactions between gender-affirming hormones and PrEP [4, 44], might also be relevant in these associations; more research is needed to explore whether this concern is related to PrEP generally, or specifically to LAI-PrEP. However, PrEP campaigns that address hesitation to use injectable PrEP among people who are less experienced with injectable medications could yield change. This could involve messages supporting overcoming a fear/aversion to needles, possibly leveraging the fact that the majority of the U.S. population has now been vaccinated for COVID-19 through injection. Some studies have shown that TWTFP express aversion to oral-PrEP due to “pill fatigue” [10], so efforts to overcome injection hesitancy may help improve PrEP coverage among sub-populations of pill-experienced TWTFP. Further research is needed to better understand hesitations TWTFP may have for injectable PrEP and strategies to overcome those hesitations, especially for TWTFP who may benefit from PrEP but face oral-PrEP uptake or adherence challenges.

Given known associations between healthcare-related stigma experiences and healthcare engagement outcomes among TWTFP, such as healthcare avoidance, reduced healthcare utilization, decreased screenings, and delayed treatment [15, 16, 45], we were surprised that gender-related healthcare stigma experiences, which are so pervasive among TWTFP [46, 47], were not related to our PrEP willingness and preference outcomes in adjusted models. Enacted gender-related healthcare stigma was associated with LAI-PrEP willingness at the bivariate level, suggesting a relationship to further explore. The null adjusted findings could be due to our sample primarily including non-Hispanic White participants; healthcare postponement due to transmisogyny and associated stigmas may be more prevalent among transgender women of color, who also face racism and other intersectional stigmas and discrimination. It is also possible that by using an attribute-specific measure of stigma (i.e., assessing stigma due specifically to gender identity) we were not able to fully capture TWTFP’s experiences and anticipation of stigma within the healthcare system, which may be attributed to a different individual social position (e.g., race, class) or may be better understood intersectionally. Stigma experiences may be difficult to disentangle and occur due to a variety of forces of marginalization including homophobia, racism, and classism. It is also possible that these measures of stigma, which focus on the interpersonal level, do not capture the experiences of structural stigma which occur within the healthcare system, and that may be integral to if and how TWTFP engage with providers. Further, the different healthcare engagement protocols required for long-acting injectable vs. oral-PrEP (e.g., 6–7 vs. 4 annual visits) may in fact become relevant for TWTFP decision making, but those differences may not have been known to the respondents at the time of survey administration. Future research should examine the relationship between PrEP healthcare engagement protocols, including the potential of telehealth PrEP visits and at-home PrEP administration, on PrEP modality willingness and preference among key populations.

### Limitations

These results should be considered in light of several limitations. First, limited guidance is available regarding which groups of transgender women are at greatest risk for acquiring HIV. Therefore, we included the entire sexually active sample (who met other analytic sample criteria). However, assuming that all sexually active TWTFP are at risk of HIV perpetuates stigma and limits our analyses and conclusions by including participants who have lower HIV risk and therefore potentially diffusing the accuracy of our findings. Additional guidance on HIV-risk among TWTFP is needed from the CDC and other public health leaders, similar to what is available for cisgender MSM [28]. Second, our sample was recruited through convenience sampling and is overwhelmingly White, which may limit the generalizability of our findings to the overall population of TWTFP, which is more racially diverse [47]. Given heightened HIV risks among transgender women of color [48], additional research is needed with Black, Hispanic, and/or other TWTFP of color. Third, our survey items for PrEP modality outcomes asked about potential initiation in the upcoming month. While this timeframe was deliberately chosen to capture intention to initiate rather than more general willingness, it is important to note that there may have been additional participants who would be interested in initiation but would need more time to act. Fourth, there may be additional relevant PrEP decision-making variables, such as the perceived importance of LAI-PrEP side effects [42] and perceived HIV risk, which we were unable to explore given available data. Future research looking at those variables, including ways they may mediate or moderate the relationships we found in the present study, could provide additional insight into PrEP interest and decision-making. Finally, this study was cross-sectional, and therefore all findings are of association and should be interpreted as such.

## Conclusions

Given that PrEP use remains suboptimal among TWTFP is the U.S., HIV prevention efforts must innovate. Expanded PrEP modality options with varying healthcare interaction protocols are one such innovation. Our study found that over a quarter of PrEP-naïve U.S. TWTFP respondents who remain at risk of HIV were willing to use LAI-PrEP, which was associated with HCP sexual health discussions, and nearly half of those willing to use multiple PrEP modalities preferred LAI-PrEP. We also found preliminary evidence suggesting further research is warranted into the relationship between past medication modality experience and LAI-PrEP willingness and preference. Currently in the U.S., stigma directed towards TWTFP is growing at both the individual and structural levels, which will negatively impact HIV prevention efforts. This includes actions at the state and federal levels to eliminate access to gender-affirming care and funding for research that acknowledges the existence of transgender people. Taken together, these results suggest that offering a range of PrEP modality options and creating and maintaining healthcare and HIV prevention spaces that are safe for discussions between TWTFP and HCPs is critical to increasing PrEP use and decreasing HIV risks.

## Supplementary Information

Below is the link to the electronic supplementary material.

Supplementary material 1 (DOCX 14.3 kb)

## Data Availability

Data cannot be shared publicly because of the sensitive nature of the data. Data are available from the first author upon reasonable request.
