# Supplementary material for: The Role of Healthcare-Related Experiences in Willingness and Preference for Long-Acting Injectable PrEP (LAI-PrEP) Among Transfeminine People in the United States
Source: AIDS Behav. 2026 Jan 27;30(9):2856–65. doi: 10.1007/s10461-025-05016-y (PMC13585847; doi:10.1007/s10461-025-05016-y)
Supplement: Supplementary file 1 [file 10461_2025_5016_MOESM1_ESM.docx]

**Supplementary Table 1: Gender-related Healthcare Stigma Items**

**Anticipated**

Have you ever felt afraid to go to health care services because you worry someone may treat you poorly because of your gender identity?

Have you ever avoided going to health care services because you worry someone may treat you poorly because of your gender identity?

Have you ever worried that a healthcare provider would judge you for your gender identity?

Have you ever worried that a healthcare provider would treat you differently if you told them about your gender identity?

Have you ever been concerned you would make a healthcare provider uncomfortable talking about your gender identity?

**Enacted**

Have you ever heard health care providers gossiping about you (talking about you) because of your gender identity?

Have you ever felt that you were not treated well in a health center because someone knew about your gender identity?

Supplementary Table 2: Sensitivity Analyses for various cut-points of anticipated gender-related healthcare stigma score variable

|  | Willing to use LAI-PrEP | | | Prefer LAI-PrEP | | |
| --- | --- | --- | --- | --- | --- | --- |
|  | aPR | 95% CI | p-value | aPR | 95% CI | p-value |
| Stratification of anticipated gender-related healthcare stigma score: |  |  |  |  |  |  |
| - Highest 33% vs. lower | 1.09 | 0.90–1.32 | 0.382 | 1.03 | 0.80–1.34 | 0.806 |
| - Highest 66% vs. lower | 1.08 | 0.90–1.30 | 0.386 | 1.11 | 0.86–1.42 | 0.428 |
| - Highest 40% vs. lower | 1.11 | 0.92–1.32 | 0.272 | 1.02 | 0.81–1.28 | 0.887 |
| - Highest 60% vs. lower | 1.09 | 0.92–1.31 | 0.320 | 1.04 | 0.83–1.31 | 0.723 |
| - Highest 25% vs. lower | 1.09 | 0.90–1.32 | 0.382 | 1.03 | 0.80–1.34 | 0.806 |
| - Highest 75% vs. lower | 1.09 | 0.90–1.32 | 0.396 | 1.04 | 0.79–1.36 | 0.790 |
| - Any anticipated gender-related healthcare stigma vs. none | 1.07 | 0.88–1.30 | 0.469 | 1.02 | 0.78–1.33 | 0.901 |
